# Prediction of off-target specificity and cell-specific fitness of CRISPR-Cas System using attention boosted deep learning and network-based gene feature

**DOI:** 10.1371/journal.pcbi.1007480

**Published:** 2019-10-28

**Authors:** Qiao Liu, Di He, Lei Xie

**Affiliations:** 1 Department of Computer Science, Hunter College, The City University of New York, New York City, NY, United States of America; 2 Ph.D. Program in Computer Science, The Graduate Center, The City University of New York, New York City, NY, United States of America; 3 Ph.D. Program in Biochemistry and Biology, The Graduate Center, The City University of New York, New York City, NY, United States of America; 4 Helen and Robert Appel Alzheimer’s Disease Research Institute, Feil Family Brain & Mind Research Institute, Weill Cornell Medicine, Cornell University, New York City, NY, United States of America; University of Trento, ITALY

## Abstract

CRISPR-Cas is a powerful genome editing technology and has a great potential for *in vivo* gene therapy. Successful translational application of CRISPR-Cas to biomedicine still faces many safety concerns, including off-target side effect, cell fitness problem after CRISPR-Cas treatment, and on-target genome editing side effect in undesired tissues. To solve these issues, it is needed to design sgRNA with high cell-specific efficacy and specificity. Existing single-guide RNA (sgRNA) design tools mainly depend on a sgRNA sequence and the local information of the targeted genome, thus are not sufficient to account for the difference in the cellular response of the same gene in different cell types. To incorporate cell-specific information into the sgRNA design, we develop novel interpretable machine learning models, which integrate features learned from advanced transformer-based deep neural network with cell-specific gene property derived from biological network and gene expression profile, for the prediction of CRISPR-Cas9 and CRISPR-Cas12a efficacy and specificity. In benchmark studies, our models significantly outperform state-of-the-art algorithms. Furthermore, we find that the network-based gene property is critical for the prediction of cell-specific post-treatment cellular response. Our results suggest that the design of efficient and safe CRISPR-Cas needs to consider cell-specific information of genes. Our findings may bolster developing more accurate predictive models of CRISPR-Cas across a broad spectrum of biological conditions as well as provide new insight into developing efficient and safe CRISPR-based gene therapy.

## Author summary

CRISPR-Cas is a powerful genome editing technology and has a great potential for *in vivo* gene therapy. To translate CRISPR-Cas into an efficient and safe therapeutic, it is critical to select target genes and design target-specific single guide RNAs such that they could maximize on-target *in vivo* efficiency as well as minimize the side effect induced by either off-target or on-target genome editing in undesired tissues. Due to experimental and clinical limitations, the CRISPR-Cas target efficiency and specificity in an intended condition (e.g. human) often need to be inferred from results in different conditions (e.g. animal model). This translational process imposes a big challenge in experimental design and potential risk in clinical development. To improve the cell-specific predictability of machine learning models and reveal important biological feature that determines the transferability of CRISPR-Cas9 across different cells, we develop an accurate and interpretable machine learning model that integrates features extracted from attention-based deep learning and knowledge-based cell-specific gene property. Our models significantly improve the performance of off-target specificity and cell-specific on-target efficiency prediction. We discover that network-based gene property is a key determinant of model predictability. Our finding may provide new insight into developing efficient and safe CRISPR-based gene therapy.

## Introduction

The clustered regularly interspaced short palindromic repeats (CRISPR)-Cas system is a powerful tool for modifying specific genome DNA targets [1–4]. CRISPR-Cas technology has drawn significant attention and is evolving rapidly because of its broad scope of applications, such as targeted mutagenesis on model organisms, knocking out or knocking in genes for gene functions clarification and epigenomic controls, delivering base editing enzyme to target site [5–8]. More importantly, it not only has been widely used to address many fundamental biological problems but also has great potential for *in vivo* gene therapy [9–11]. For example, a mutation in the sickle cell disease (SCD) HBB gene for adult β-globin protein is corrected by CRISPR-Cas9 when the mutation is targeted in Human induced pluripotent stem cells (iPSC) [12]. The modification of the mutated exon 23 in the DMD gene improves failed muscle function in the *mdx* mouse model [13, 14]. Compared with another promising gene therapy approach RNAi, the CRISPR-Cas9 could be used for both non-permanent gene silencing and also permanent gene knockout. Besides, the CRISPR-Cas suffers less off-target effects [15]. However, many investigations are still being actively conducted on solving safety and efficiency concerns, including off-target side effects, cell fitness, *in vivo* delivery methods, control of repair mechanisms and system efficiency [16–21]. We here focus on using computational tools to optimize sgRNA design to improve sgRNA efficiency and specificity. This work can help to solve the safety challenges for the realization of CRISPR-Cas gene therapy usage due to off-target side effects and cell fitness. Moreover, because current CRISPR-Cas *in vivo* delivery methods are not tissues-specific, CRISPR-Cas could also lead to on-target side effects, due to genome editing in non-culprit or undesired tissues. We also devote our attention to designing a cell-specific prediction method.

According to current understanding, the targeting efficiency and specificity of CRISPR-Cas primarily depend on the sequence of single-guide RNA (sgRNA) as well as the local 3D structure and functional state of the target genome. For instance, the targeting process in the CRISPR-Cas system with *S*. *pyogenes* Cas9 has three fundamental requirements [6, 22]. First, the single-guide RNA (sgRNA) sequence needs to be complementary with its targeting genome sequence. Second, a Protospacer Adjacent Motif (PAM) needs to locate around the targeted site [6, 23, 24]. Finally, the off-target effect, which is caused by binding sgRNA with genome sequences that are similar to the targeting sequence, needs to be minimized [19, 25, 26]. These are necessary for an efficient system but are not sufficient. Other local structural factors were also proposed to affect sgRNA targeting efficiency and specificity [25, 27]. For instance, Open chromatin sites may promote sgRNA binding due to their high accessibility, and DNase sensitivity data provide information on the chromatin coverage state and target sites accessibility [28, 29]. Besides, to understand the fitness of cells after treatment, we have to investigate the cellular response to the edited genome. The cellular response depends on the distinct molecular contents of the cell. For example, the same sgRNA could cause different ultimate cellular responses in different cells. Thus, cell-specific features which can illustrate the role of a gene in a systematic view are desired to be incorporated to predict the cellular response. Gene expression profiles illustrate the cell-specific molecular context and thus could be taken into consideration. The property of the target gene in the gene-gene interaction network may provide its global context in a cell. To our knowledge, no computational analysis has included cell-specific information into the cellular response prediction of CRISPR-Cas system.

Nowadays, computational analysis plays a vital role in sgRNA design. A wealth of system-level omics data have been collected using high-throughput CRISPR-Cas screening and next generation sequencing [30, 31]. Despite the considerable success of existing machine learning models trained with these large scale dataset (e.g. [27, 32, 33]), sgRNA targeting efficiency and specificity prediction is still a challenging problem, and few of these models take the cell-specific information into account. Here we present innovative prediction models for sgRNA off-target specificity and on-target efficiency prediction. Our studies made several seminal contributions. First, we develop two novel machine learning models: AttnToMismatch_CNN and AttnToCrispr_CNN, which take advantage of the most successful deep learning architectures for sequential analysis: attention-based transformer [34–36]. Second, we for the first time, incorporate cell-specific network-based gene property into the models. Third, we develop a method to encode a sgRNA sequence as a novel matrix representation. Fourth, we implement a universal feature ranking algorithm for the deep learning models to determine the feature importance. Finally, our models can be applied to both CRISPR-Cas9 and CRISPR-Cas12a systems. With these merits, the AttnToMismatch_CNN model significantly outperforms state-of-the-art models for off-target sgRNA specificity prediction in both CRISPR-Cas9 and CRISPR-Cas12a datasets. AttnToCrispr_CNN also shows competitive performance on on-target efficiency prediction, especially on negative selection experiment dataset. Moreover, we demonstrate that the network-based gene property significantly improves post-treatment cell-specific cellular response prediction for negative selection experiment, which is a more suitable setup for cell fitness study. Additionally, the feature importance study provides new biological insight for the prediction of sgRNA targeting efficiency and specificity.

## Results

### Overview of AttnToMismatch_CNN, AttnToCrispr_CNN, and seqCrispr model architectures

**Fig 1:**
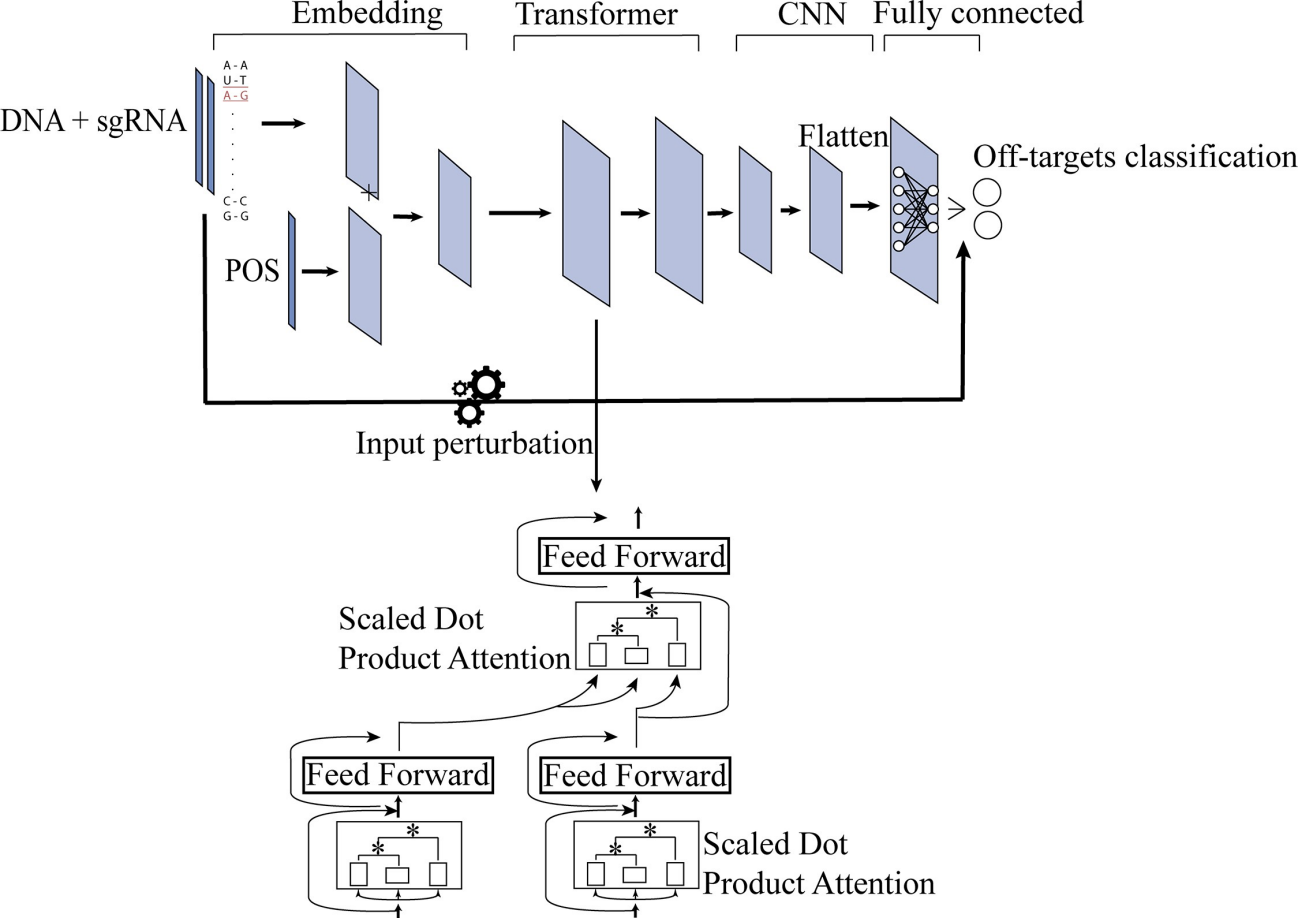
Schematic representation of the off-target specificity prediction model, AttnToMismatch_CNN.

Given that the CRISPR-Cas system is a potential gene therapy technique, off-target specificity is a critical issue for safety purpose [19, 20, 26, 37]. For the off-target specificity prediction, we implemented a deep neural network AttnToMismatch_CNN, which is comprised of four components (Fig 1). The first component is an embedding layer. Each base from sgRNA and its counterpart in DNA compose an aligned base pair. Each base pair is encoded as a vector representation. In turn, the aligned sgRNA and DNA sequences are encoded into a matrix. Besides, a positional embedding layer encodes each position into a vector. Then all positional vector representations are concatenated together to output a matrix for the aligned sequence. The base-pair and positional matrices are elementwise added. With this embedding method, the base pairs at different positions are encoded into distinct vector representations. The output of the embedding layer flows into the second component, a transformer layer. This module has shown superior performance on sequential analysis, especially in the natural language processing field [34–36]. The transformer is composed of an encoder part and a decoder part. Both encoder and decoder have multiple multi-heads scaled dot product based attention modules sequentially connected. The output of the transformer has the same dimension with its input and subsequently flows into the third component: a convolutional neural network layer (CNN). CNN comprises two Conv2d layers and two Maxpooling layers interleaved with each other. The last component is a Fully connected layer. The output from CNN is flattened and flows into the fully connected layer, which includes a softmax function to predict the probability of a sgRNA to be positive samples or negative samples.

**Fig 2:**
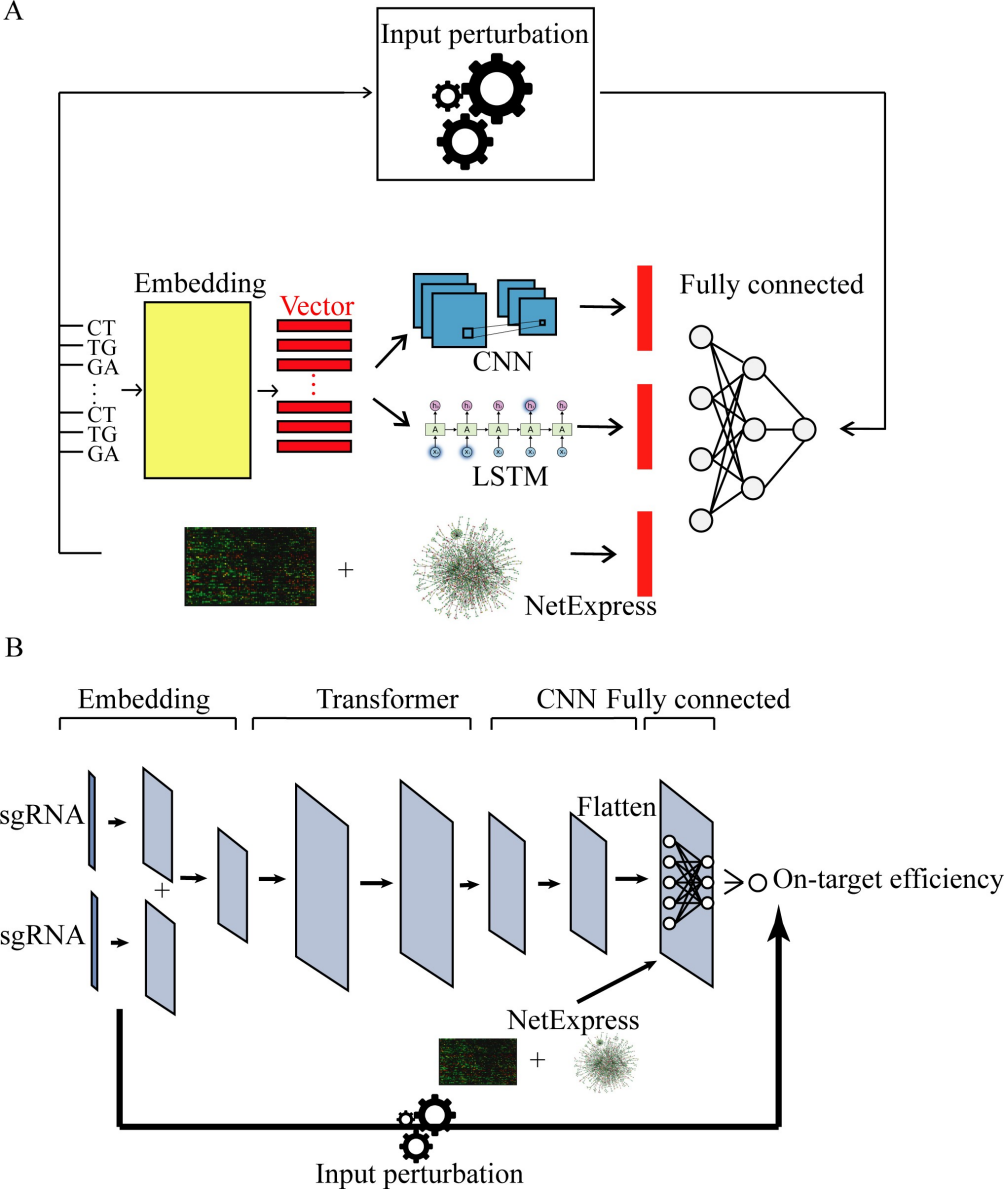
**Schematic representation of on target efficiency prediction models, A) SeqCrispr and B) AttnToCrispr_CNN**.

For on-target efficiency predictions, we implemented two models, seqCrispr and AttnToCrispr_CNN (Fig 2A and 2B). Both of them are deep neural network models that consist of four components. The main differences between them are their second and third components. seqCrispr harbors a long short term memory (LSTM) component and CNN component in parallel, while AttnToCrispr_CNN has a Transformer component followed by a CNN component. Both LSTM and transformer are popular and successful modules used to analyze sequential data in the natural language processing field. However, the transformer has shown better performance than LSTM [38, 39]. The CNN, LSTM, and transformer component enable the overall model to learn the interaction of a base in the sequence with not only proximal bases but also other distant bases. i) In seqCrispr, the first component is an embedding layer. A sliding window of length 2 was used to extract dimer from each position. For example, the 3rd dimer is a 2-bases sequence located from position 3 to position 4. Each dimer is encoded as a vector representation, and all dimer vectors in a sequence can be concatenated to a matrix as the representation for the sequence. The output of the embedding layer flows into both CNN and LSTM layers in parallel. The output from these two layers is flattened and concatenated together with optional biological features. The last fully connected layer has a linear regression layer after all, to output an on-target efficiency score. ii) In AttnToCrispr_CNN, dimers are also extracted with a sliding window of length 2-bases. Same as AttnToMismatch_CNN, a sequence will be encoded to a matrix and elementwise summated with positional embedding matrix to generate the eventual embedding matrix. The embedding matrix will be the input of the second component, a transformer layer. The third and fourth components are the same as those of AttnToMismatch_CNN except that AttnToCrispr has a linear regression layer as the final output. Besides, on top of all these infrastructures, input perturbation method is implemented to study the feature importance [40].

### AttnToMismatch_CNN model significantly outperforms state-of-the-art models on off-target specificity prediction

**Fig 3:**
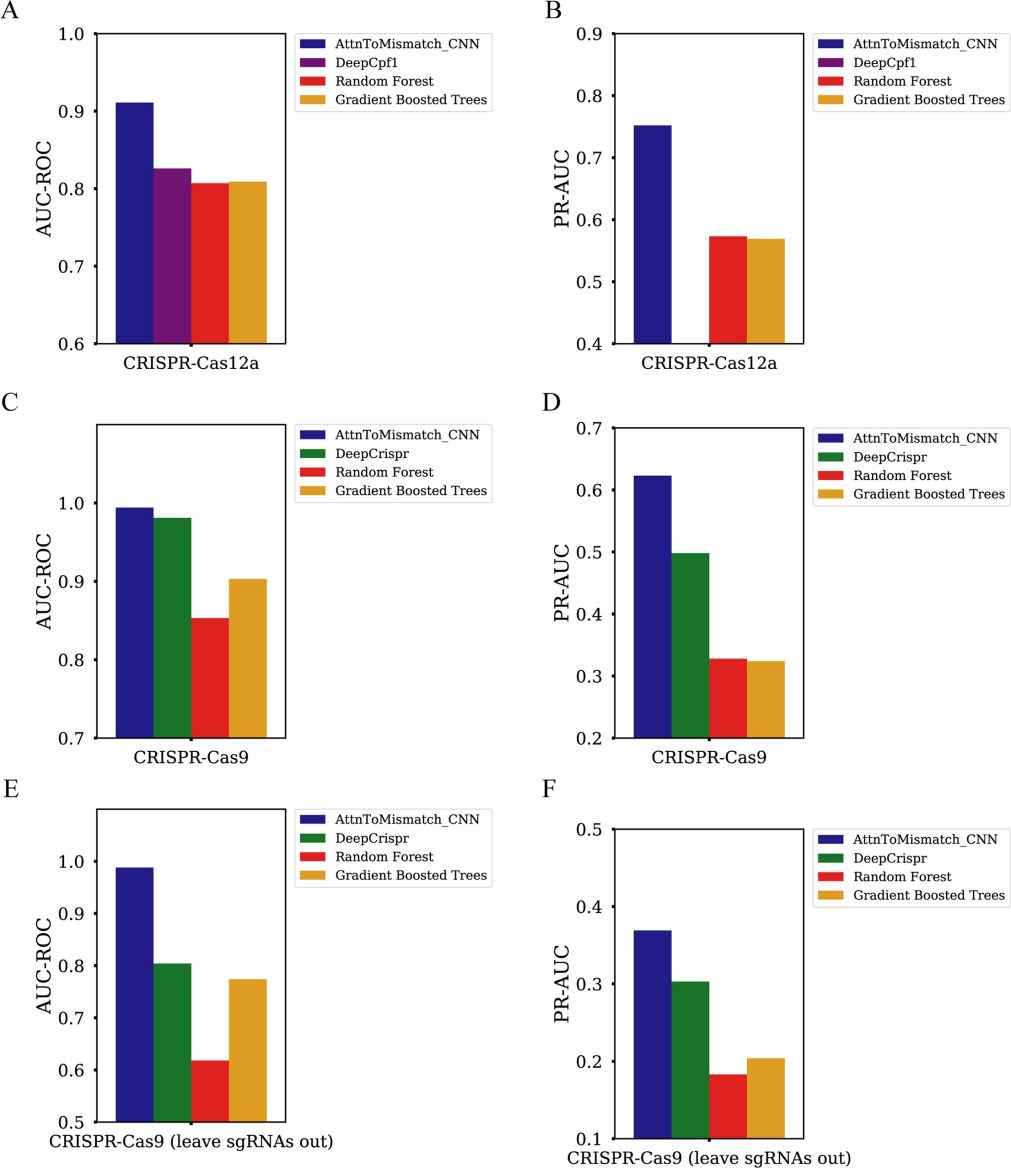
Performances comparison of off-target specificity prediction models, including AUC-ROC and PR-AUC scores of AttnToMismatch_CNN, DeepCpf1, DeepCrispr, Random Forest, and Gradient Boosted trees models in 2 different scenarios. A)-B) Crispr-Cas12a (Crispr-Cpf1). DeepCpf1 PR-AUC score is not provided in the previous study [33]. C)-F) Crispr-Cas9. C) and D) are the performances with the 5-fold cross-validation method. E) and F) are the performances by leaving three sgRNAs out as test dataset (leave-sgRNAs-out). In B), the PR-AUC of DeepCpf1 was not reported.

To evaluate the performance of the AttnToMismatch_CNN model, we tested it with two independent published dataset and compared its performance with state-of-the-art models: deepCpf1 [33]and deepCrispr [32]. Because CRISPR-Cas12a and CRISPR-Cas9 are the two most popular genome editing tools, we selected data which were collected with these two techniques. To keep consistent with deepCpf1 study, we used the same setup as theirs, including the followings: 1) Both of two input sequences have 27 nucleotides. The 4-bases PAM sequence is at the 5’end of spacer. 2) We sorted the sgRNA-DNA pairs based on their indel frequencies in ascending order. Then we labeled the top 20% sgRNA-DNA mismatch with highest indel frequencies as positive samples and the remaining as negative samples. 3) The performance was tested with 5-fold cross-validation. We compared our model performance with three other models, Random Forest, Gradient Boosted Trees, and deepCpf1. Random Forest and Gradient Boosted Trees are two conventional machine learning models that have shown superior performance in many biological applications compared to other machine learning models. deepCpf1 is a deep neural network mainly based on convolutional neural network. It is the state-of-the-art deep learning models on predicting off-target specificity in CRISPR-Cas12a system [33]. The main differences between deepCpf1 and AttnToMismatch_CNN come from the facts: AttnToMismatch_CNN has an extra Transformer layer in front of CNN, and uses an embedding layer to learn the vector representation for a base pair, while deepCpf1 employs a one-hot encoding strategy. AttnToMismatch_CNN significantly outperforms other models by a margin of more than 10% when the performance are evaluated by the AUC-ROC and PR-AUC metrics (Fig 3A).

Then we evaluated AttnToMismatch_CNN off-target specificity prediction performance on CRISPR-Cas9 dataset. We also keep the same setup with deepCrispr study [32]. 1) Around 165,000 negative samples and 656 positive samples are included in the dataset. 2) Different from CRISPR-Cas12a system, spacer in the CRISPR-Cas9 system has 20 nucleotides, and a 3-bases PAM sequence locates at its 3’end. 3) Because this dataset is highly imbalanced, we oversample positive samples for each mini-batch during the training process so that each batch has a similar amount of negative samples and positive samples. Details of implementation are described in the Methods section. We firstly tested Random Forest, Gradient Boosted Trees, deepCrispr, and AttnToMismatch_CNN models using 5-fold cross-validation method. deepCrispr is the current state-of-the-art deep learning model for the CRISPR-Cas9 system on-target specificity and efficiency prediction. We used 80% samples as training data and the remaining 20% samples as test data. AttnToMismatch_CNN shows superior performance on both AUC-ROC and PR-AUC scores (Fig 3B and 3C). It is worth noting that PR-AUC is believed to be a more suitable metric applied to test models performance on imbalanced dataset [25]. In deepCrispr dataset, the number of negative samples is much larger than that of positive samples. When the number of false-positive samples increases, the false positive rate would not change too much due to a large number of negative samples. However, the precision, which is the fraction of the number of false-positive samples over the number of predicted positive samples, is more sensitive to the increase of false-positive samples. In such a situation, PR-AUC is a more meaningful metric in model performance assessment. In the 5-fold cross-validation scenario, AttnToMismatch_CNN outperforms other models by around 20% on PR-AUC score (Fig 3B). We then compared the performance of these models in a more rigorous condition. We selected three sgRNAs and excluded them from the training process. Then we tested models performance with these three sgRNAs data. AttnToMismatch_CNN also achieves better performance than other models by 20% for both AUC-ROC and PR-AUC metrics (Fig 3D and 3E).

### NetExpress score contributes significantly to the overall cellular response prediction of CRISPR based genome editing

Cellular responses following genome editing should be carefully considered for the utilization of CRISPR based gene therapy. We carefully curated CRISPR-Cas9 experiment data from published literature in three cell lines, K562, A549, and NB4 [41, 42]. All these data were collected with CRISPR-Cas9 based genome-wide negative selection approach. In these experiments, when edited or loss-of-function genes are essential for cell growth or proliferation, these cells tend to die, and the number of cells may decrease. Therefore, the change of cell counts before and several days after importing CRISPR-Cas9 system to cells indicates the overall cellular response due to potential genome editing. We used the log2 fold change (log2fc) of sgRNAs for the following analysis because sgRNA counts change can indirectly indicate the cell counts change. We first filtered out approximately 4,500 sgRNAs, which were found in the data from all three cell lines, and noticed that the spearman correlation of log2fc for these sgRNAs among different cell lines are 0.37, 0.45 and 0.48 (Table 1). It proves that the same sgRNA would cause significant different cellular responses in different cell lines. The differences could be attributed to cell-line specific cellular composition, batch effects, or random errors. In the following study, we focus on exploring cellular response differences caused by cell-line specific cellular composition.

**Table 1:** Spearman correlation of around 5,000 sgRNAs corresponding log2fc values in K562, A549, and NB4 cell lines. These sgRNAs were used in all three cell lines.

Cell lines in comparison	Spearman correlation
K562-A549	0.454
A549-NB4	0.482
K562-NB4	0.370

**Fig 4:**
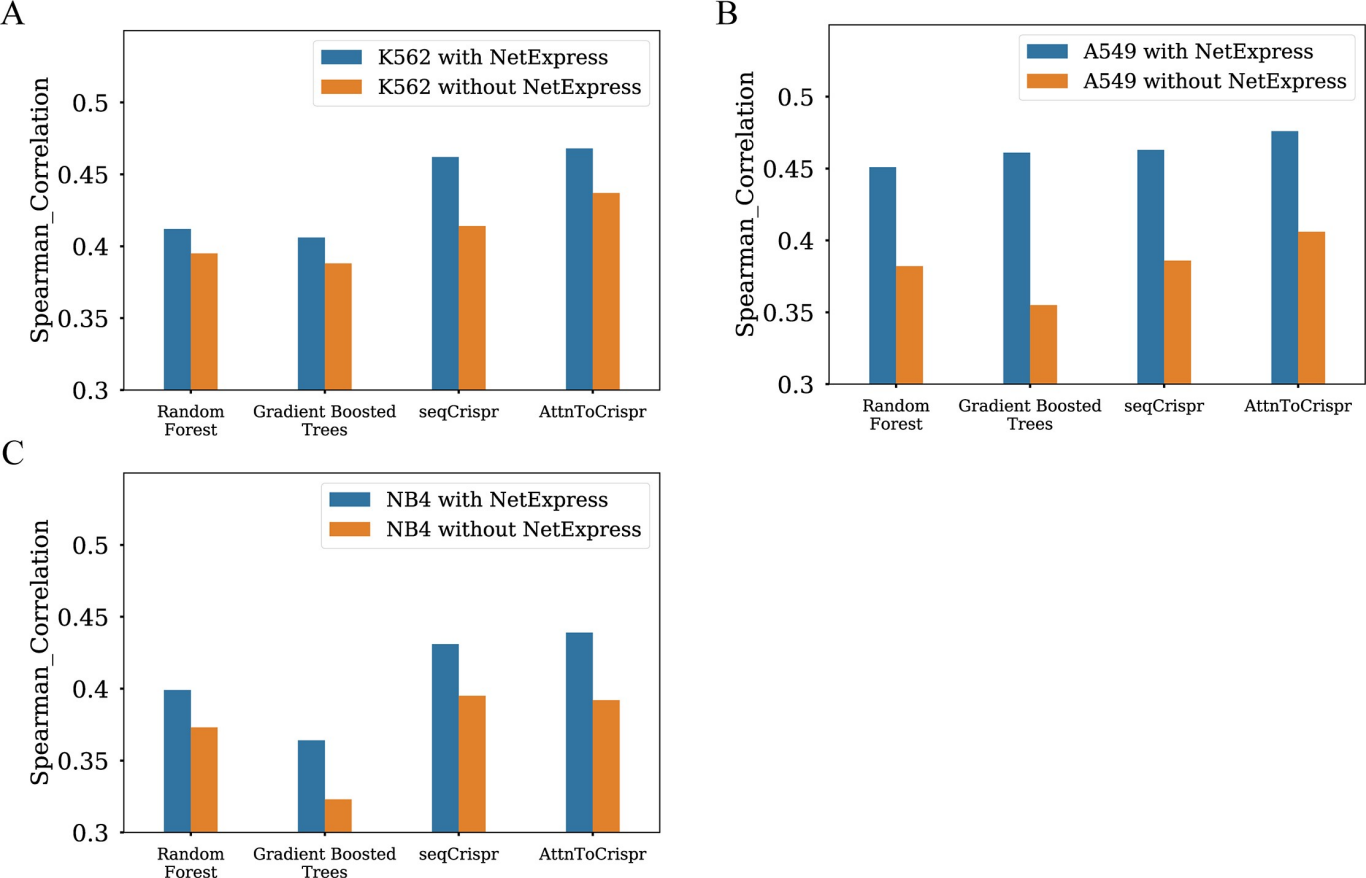
Performance comparison of models that are trained with or without NetExpress score. Spearman correlation metric is measured to test models performance on three negative selection experiment datasets. A) K562 cell line dataset. B) A549 cell line dataset. C) NB4 cell line dataset.

We trained predictive models, Random Forest, Gradient Boosted Trees, SeqCrispr, and AttnToCripsr_CNN. The ultimate output of these models is log2fc of sgRNA counts. In order to make the cell-specific cellular response prediction, we included a quantitative score to represent cell-specific gene property. This score is termed as NetExpress, which is derived from both cell-line specific gene expression profile and gene-gene interaction network (details in Methods) [23]. It is the summation of weighted gene expression values of a gene’s neighbor genes in the gene-gene interaction network. Intuitively, NetExpress score can be interpreted as the gene importance score in system-level gene-gene interaction network given the context of whole genome-wide gene expression profiles. We then tested the models’ performance with input features, including or excluding the NetExpress score. The other features are the sgRNA sequence feature and Copy Number Variation. All models with NetExpress scores outperform others without this feature by 2%-15% (Fig 4A, 4B and 4C and S1 Table). This result suggests that the network-based gene property improves cellular response prediction of CRISPR caused genome editing, at least for these negative selection dataset. Again, AttnToCrispr outperforms seqCrispr, and both of them are superior to Random Forest and Gradient Boosted Tree, as shown in Fig 4.

### AttnToCrispr_CNN has superior performance for CRISPR on-target efficiency prediction

To evaluate our model performance for on-target efficiency prediction, we compared seqCrispr and AttnToCrispr_CNN with two state-of-the-art deep learning models, deepCpf1 on CRISPR-Cas12a dataset and deepCrispr on CRISPR-Cas9 dataset. These datasets were selected because they both have more than 15,000 CRISPR sgRNA samples. CRISPR-Cas12a datasets were generated in one cell line. sgRNAs target multiple genes at wide-spread locations of the genome. To have an apple-to-apple comparison with deepCpf1, we used the same training dataset and test dataset. sgRNA’s indel frequencies were used as its ultimate output. The main difference between deepCpf1 and AttnToCrispr_CNN originates from that AttnToCrispr has an extra Transformer component between the embedding component and CNN (Fig 2). As suggested and used in previous on-target efficiency prediction works, Spearman correlation is a more appropriate metric for this regression problem [25]. Besides, we also included the Pearson correlation and MSE to give a comprehensive performance evaluation. AttnToCrispr_CNN has a better performance than deepCpf1 on CRISPR-Cas12a sgRNA on-target efficiency prediction on all metrics (Table 2). Different from off-target specificity prediction model AttnToMismatch_CNN, the input sequence length used in AttnToCrispr_CNN model is 34 bases. Besides, we also compared the performance of models using different sgRNA sequence lengths and confirmed that 34 bases gave the best performance, which was also mentioned in the deepCpf1 study [33].

**Table 2:** Comparison of models performances on Crispr-Cas12a (Crispr-Cpf1) dataset. Spearman correlation, Pearson correlation, and mean squared error metrics (MSE) are compared.

Dataset	Model	Spearman	Pearson	MSE
Cas12a	Random Forest	0.643 ± 0.002	0.648 ± 0.002	578 ± 63
	Gradient Boosted Trees	0.684 ± 0.002	0.677 ± 0.002	549 ± 57
	deepCpf1	0.760	-	435
	seqCrispr	0.765 ± 0.005	0.760 ± 0.004	442 ± 33
	**attnToCrispr_CNN**	**0.778 ± 0.003**	**0.781 ± 0.003**	**412 ± 27**

The CRISPR-Cas9 dataset was curated from three different studies and in four cell lines, HCT116, HL60, HEK293T, and HeLa [25, 43, 44]. These data were utilized for training deepCrispr model on sgRNA on-target efficiency prediction [32]. The normalized on-target efficiency scores were calculated with log2 fold change, which indirectly reflects the abundance changes of cells before and several days after treatment with the CRISPR-Cas9 system having a specific sgRNA. Importantly, data in HCT116, HL60, and HeLa were generated with high-throughput negative selection screening. These sgRNAs targeted hundreds of genes. On the other side, positive selection experiment dataset was obtained in HEK293T cell line, in which only eight genes were targeted. However, the cellular responses arise from targeted genes function on a given selective pressure, like a drug, in the positive selection experiment. We firstly compared model performances in a more rigorous circumstance, where the data in three cell lines were used for training purpose, and the last cell line was kept unseen during training. AttnToCrispr_CNN performance is higher than other models when the data in either HCT116, HL60, or HeLa cell lines are left out (Table 3). It is worth mentioning that we hardly see any correlation between ground truth on-target efficiency scores and the predictions when we left HEK293T cell line out as test data. A similar result was also noticed in deepCrispr study. It suggests that any information of a model, which is trained with negative selection data, is hardly transferrable to the model for the prediction of positive selection effects. We also assessed AttnToCrispr_CNN with 5-fold cross-validation method. We firstly performed analysis with only negative selection dataset for two reasons: i) Our main goal is to study the cellular response following CRISPR triggered genome editing in general, not in the presence of other external factors, like drugs. ii) The leave-cell line-out tests showed that the data between negative selection dataset and positive selection dataset have scarce transferable information. We included 80% data from each cell line in the training process and the remaining 20% data were used in the testing stage. All Spearman correlations are higher than those of deepCrispr (Table 4). Besides, we also show that AttnToCrispr_CNN has superior performance on the 5-fold cross-validation test with both negative selection data and position selection data (S2 Table). Analysis of positive selection dataset was also performed to give a comprehensive overview (S3 Table).

**Table 3:** Comparison of model performances on Crispr-Cas9 dataset. Model performance was evaluated with the leave-one cell line-out method. Spearman correlation, Pearson correlation, and MSE metrics are compared.

Dataset	Cell line	Model	Spearman	Pearson	MSE
deepCrispr (leave cell line)	HL60	deepCrispr	0.25	-	-
		attnToCrispr_CNN	**0.286 ± 0.000**	**0.276 ± 0.000**	**0.0121 ± 0.0000**
	HCT116	deepCrispr	0.761	-	-
		attnToCrispr_CNN	**0.801 ± 0.000**	**0.797 ± 0.000**	**0.0006 ± 0.0000**
	HeLa	deepCrispr	0.541	-	-
		attnToCrispr_CNN	**0.591 ± 0.000**	**0.591 ± 0.000**	**0.0221 ± 0.0000**
	HEK293T	deepCrispr	**0.069**	-	-
		attnToCrispr_CNN	-0.017 ± 0.001	-0.013 ± 0.001	0.384 ± 0.0022

**Table 4:** Model performances of negative experiment data in HL60, HCT116, and HeLa cell lines with 5-fold cross-validation.

Dataset	Cell line	Model	Spearman	Pearson	MSE
deepCrispr (5 fold cv)	HL60	deepCrispr	0.262	-	-
		attnToCrispr_CNN	**0.406 ± 0.000**	**0.377 ± 0.000**	**0.0146 ± 0.0000**
	HCT116	deepCrispr	0.654	-	-
		attnToCrispr_CNN	**0.698 ± 0.000**	**0.713 ± 0.000**	**0.0112 ± 0.0000**
	HeLa	deepCrispr	0.501	-	-
		attnToCrispr_CNN	**0.573 ± 0.000**	**0.566 ± 0.000**	**0.0241 ± 0.0000**

### Input perturbation based feature importance analysis reveals biological insights

**Fig 5:**
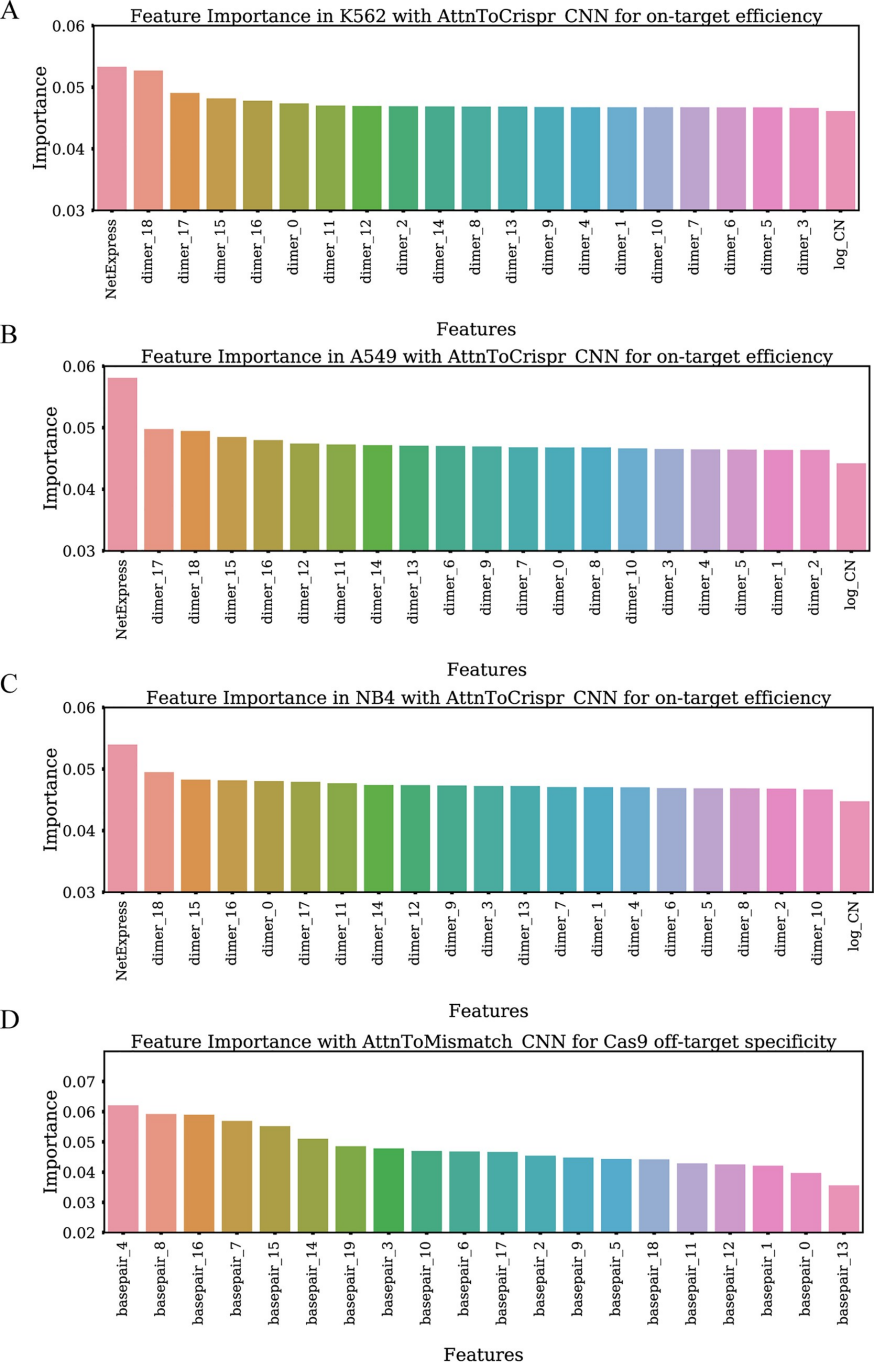
Feature importance study with input features perturbation method. The feature importance of AttnToCrispr_CNN on-target efficiency prediction model, which is trained with Crispr-Cas9 dataset in A) K562 cell line B) A549 cell line and C) NB4 cell line. Each dimer is two contiguous nucleotide bases on the input sequence. D) The feature importance of AttnToMismatch_CNN off-target specificity prediction model, which is trained with Crispr-Cas9 dataset. Each dimer is a nucleotide base pair, with one from a sgRNA and its counterpart in the target DNA.

**Fig 6:**
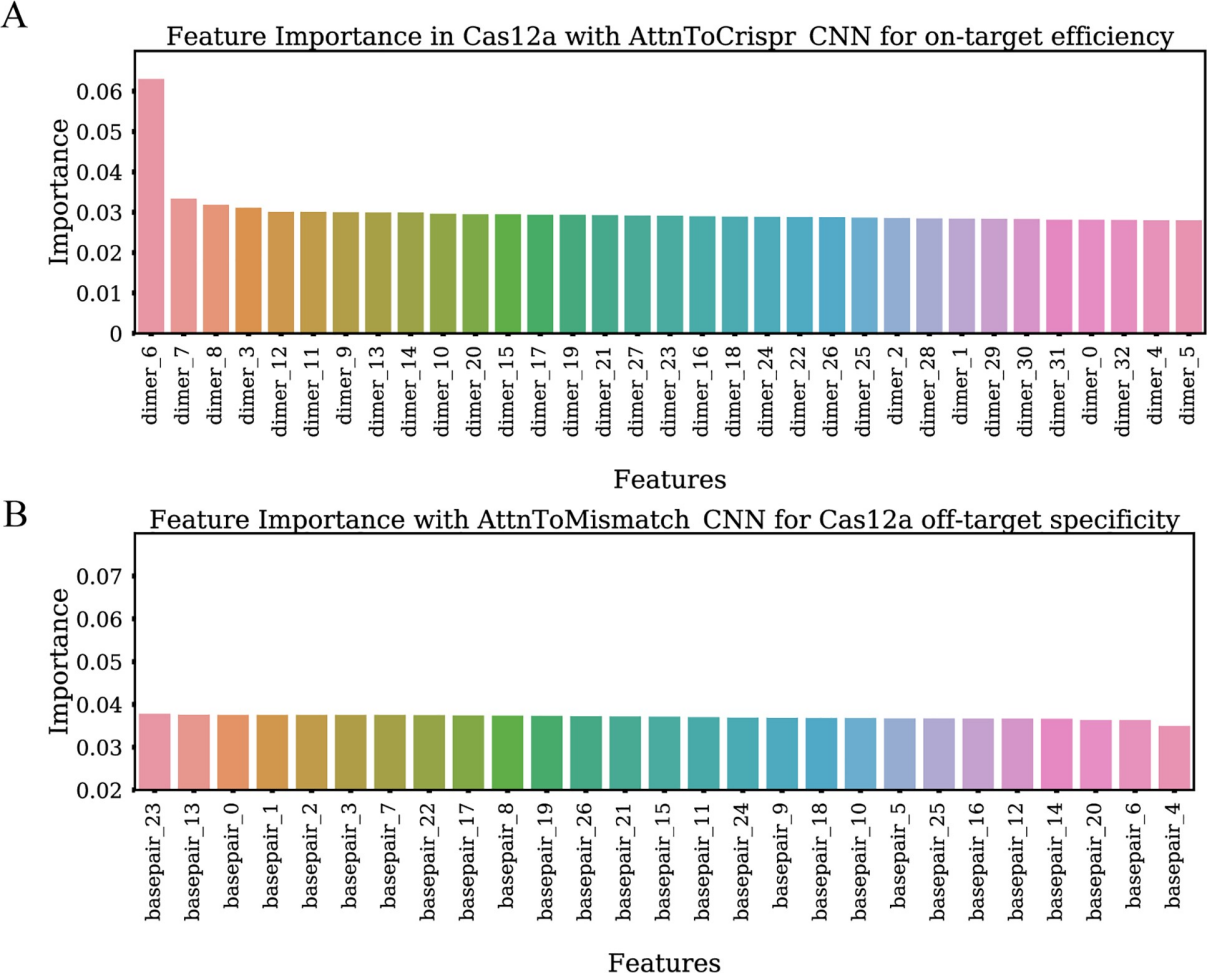
Feature importance study with input features perturbation method. A) The feature importance of AttnToCrispr_CNN on-target efficiency prediction model, which is trained with Crispr-Cas12a dataset. Each dimer is two contiguous nucleotide bases on a input sequence. B) The feature importance of AttnToMismatch_CNN off-target specificity prediction model, which is trained with Crispr-Cas12a dataset. Each dimer is a nucleotide base pair, with one from a sgRNA and its counterpart in the target DNA.

We incorporate an input perturbation component into our deep neural network models in order to explore feature importance. In this algorithm, feature importance was determined by perturbating each input feature across all samples and examine the decline in models final performance. 1) We checked feature importance for on-target efficiency prediction model AttnToCrispr_CNN with CRISPR-Cas9 data in K562, A549, and NB4 cell lines. It shows that NetExpress score is the most important input feature (Fig 5A, 5B and 5C). Dimer_18, which is the 19th -20th bases in sgRNA, contributes significantly to these models. The indexing of dimer starts from 0, and its direction is from sgRNA 5’end to its 3’ end. This observation is consistent with the experimental discovery that unwinding of target site dsDNA starts from the 3’ end of sgRNA [45]. It implies that the initialization of the unwinding process is critical for efficient sgRNA targeting. 2) The feature importance study for CRISPR-Cas9 off-target specificity prediction illustrates that dimer_0 and dimer_1 are less critical than other dimers (Fig 5D). This result confirms that the mismatches in the first and second positions of 5’ end of sgRNA are highly tolerable. 3) The first two most important features of On-target efficiency prediction model AttnToCrispr_CNN for CRISPR-Cas12a is dimer_6 and dimer_7, which locates in the PAM region (Fig 6A). 4) The feature importance of each input feature in off-target specificity prediction model AttnToMismatch_CNN does not show a notable difference from each other. However, we can not rule out the possibility that these less significant differences are owing to the limited amount of data in the dataset (Fig 6B).

## Discussion

The successful computer-aided design of sgRNAs could save days of work and the cost of experimental reagents [46] as well as reduce the potential risks in the clinical trial. Training a reliable deep learning model typically requires high-quality and large-scale dataset. Fortunately, many large-scale CRISPR-Cas experimental datasets are produced with genome-wide CRISPR-Cas screening, in combination with next generation sequencing technique. Machine learning models, particularly deep learning, can be then used to build predictive models using these datasets, and have shown to be successful in optimizing the sgRNA design [25]. For example, both deepCrispr and deepCpf1 optimized sgRNAs design on CRISPR-Cas9 and CRISPR-Cpf1 system, respectively [27, 32, 33]. Deep learning models show superior performance to conventional machine learning models. However, most of the deep learning models are still black boxes. One of the critical impediments for deep learning is to interpret the importance of input features of the model. Our input features perturbation method could be easily adapted to all deep learning models for input features importance analysis and thus could be one of the solutions of this technical issue.

One major obstacle when considering the application of the CRISPR-Cas system in gene therapy is the potential off-target effect. AttnToMismatch_CNN significantly outperforms current state-of-the-art models with various evaluation metrics on sgRNA off-target specificity prediction. It improves the true positive rate and in the meantime, leads to a noticeable reduction in the false positive rate, which is a challenging task for highly imbalanced dataset. In practice, the improvement in the prediction of sgRNA specificity will save time and cost on exploring false-positive off-target sites. Moreover, our results strongly suggest that, given sufficient data, AttnToMismatch_CNN can be applied to study different CRISPR-Cas system for exceptional performance, as demonstrated in both CRISPR-Cas12a and CRISPR-Cas9 systems. The performance improvement comes from the introduction of two components in AttnToMismatch_CNN, embedding layer and transformer layer. The idea of encoding the extracted sequence features into vector representations is inspired by the word embedding technique [47, 48]. Many state-of-the-art models in natural language processing field are also built on top of these two components [49, 50]. The success of AttnToMismatch_CNN suggests that more advanced natural language processing technique can be used on DNA or RNA sequence analysis.

AttnToCrispr_CNN also takes advantage of the aforementioned deep learning techniques and demonstrates competitive performance on sgRNA efficiency prediction. More importantly, cells fitness concern after treatment with CRISPR-Cas system is another issue to be tackled for gene therapy application in addition to off-target side effect [51, 52]. The negative selection experiment data is a valuable resource to study the CRISPR-Cas system effect on the cellular response, specifically on cell growth. Our result suggests that the network-based gene property, which integrates information from gene-gene interaction network and gene expression profiles of neighbors of the target gene, can be a determinant predictor on the ultimate cellular response to the treatment on the target gene. The rationale is that the cellular response to the CRISPR-Cas system perturbation is a systematic response from many related genes or pathways. Gene-gene interaction network is a powerful tool to study these responses in a cellular context. Moreover, this property takes cell-specific gene expression profiles into account, and in turn, models with this property can output the prediction of cellular response based on the cell type. Our results suggest a potential way to look into the undesired tissues on-target side effect problem. Besides, the model built with other cell-specific local genetic features, including DNase-seq, Chip-seq for CTCF and H3K4me3 and RRBS data, barely shows substantial performance improvement compared to the model without these features (S4 Table). We notice a contradiction with some previous studies [27, 32]. One reason could be that the epigenomic data might not accurately illustrate the features of cells used for CRISPR-Cas system data collection because they were collected in different laboratories.

Our model significantly improves the accuracy on the off-target specificity and pioneers the cell-specific fitness prediction, which are related to safety concerns of the CRISPR-Cas system as gene therapy [51, 52]. With optimal sgRNA design, many challenges remain to be solved to maximize the genome-editing efficacy of the CRISPR-Cas system in many aspects, such as genome repair mechanisms and *in vivo* delivery of CRISPR-Cas components. All current methods have pros and cons. Non-homologous end-joining (NHEJ) and homology directed repair (HDR) are the two primary gene repair mechanisms. As a more desirable gene repair mechanism, HDR can correct culprit genome more precisely with a homologous DNA template but suffers low efficiency [51, 53, 54]. On the other side, NHEJ can repair damaged genome more efficiently but leads to various undetermined mutations, insertion, or deletion [55]. Three viral *in vivo* delivery methods being investigating are utilizing adenoviral, lentiviral, and adeno-associated viral (AAV) vectors [16, 56–58]. Despite the advancement of these methods in recent years, their usages are still impeded by different kinds of limitations [56–58]. For instances, AAV can only delivery CRISPR-Cas system components into cells safely and efficiently, but the cargo can only be a small size (<4.7kb) exogenous genome [16, 56].

On top of these, the translatability of researches *in vitro* or *ex vivo* to *in vivo* gene therapy still raises many questions. First, the recent most popular *in vivo* delivery methods are intramuscular, intraperitoneal, or intravenous injections. These are not tissue-specific methods. For example, an intravenous injection of an AAV vector carried CRISPR/Cas9 system targeting on HIV-1 causes genome cleavages in bone marrow, liver, brain, colon, spleen, heart, and lung tissues of the mouse model [59]. Thus a more tissue-specific *in vivo* delivery technique is desired such that tissues distribution affected by the CRISPR-Cas system can be well controlled. Second, given the more complicated microenvironment *in vivo*, the ultimate treatment effect was unclear and remains to be defined. The proportion of cells with edited genome can either increase or decrease after treatment based on whether the cells gain growth advantage. For this reason, our study focused on the ultimate cell-specific cellular response after genome editing by the CRISPR-Cas system in negative selection dataset. Third, the application of gene editing technique on germline faces ethic controversy. A germline CCR5 gene change using CRISPR-Cas technique by He et al. arouses concerns for its future usage and might obstruct CRISPR-Cas as gene therapy [60]. However, we believe that computational analysis has the potential to facilitate the final clinical usage of CRISPR-Cas in many perspectives. For example, repaired DNA after CRISPR-Cas mediated breaks shows specific nonrandom modification patterns [61]. With the availability of increasing experimental data and advance of computational biology techniques, these repair mechanisms can be more clear.

## Methods

### Dataset

#### Off-target dataset

In the off-target specificity prediction study, two independent datasets were used to test model performance. 1) CRISPR-Cas12a (CRISPR-Cpf1) dataset. This dataset was collected by Kim et al. (2018) [27] and was used to train deepCpf1 model by Tan et al. (2019) [33]. For comparison purpose, we applied the same labeling strategies as deepCpf1 to assign the top 20% active (high indel frequency) mismatched sgRNA-DNA sequences pairs as high activity samples or positive samples and the remaining as low activity samples or negative samples. 2) CRISPR-Cas9 dataset. 656 off-target sites were collected in multiple studies with different whole genome off-targets screening techniques across two cell lines, K562 and HEK293T [32, 62–67]. We labeled these sgRNA-DNA mismatched pairs as positive samples. These off-target sites are the same as that used in deepCrispr [32]. To collect negative samples, we used Cas-OFFinder to find potential sgRNA-DNA mismatch pairs in the whole genome where mismatched bases in each pair are less than or equal to 6 [68]. Around 165,000 negative samples are found totally.

#### On-target dataset

In the on-target efficiency prediction study, three independent datasets were utilized. 1) CRISPR-Cas12a (CRISPR-Cpf1) dataset. Kim et al. (2018) used this dataset to train an on-target efficiency prediction model deepCpf1 with deep learning technique [27]. Training dataset has 15,000 sgRNAs, and test data has 1,292 sgRNAs. Each sgRNA’s indel frequency is used as its on-target efficiency score. 2) CRISPR-Cas9 negative selection dataset. This dataset was carefully curated from previously published literature [41, 42]. Around 105,000 sgRNAs, 74, 000 sgRNAs and 74,000 sgRNAs were studied in K562, A549 and, NB4 cell lines, respectively. All these data have the following features available: copy number variation and gene expression data used for NetExpress score calculation. In these negative selection experiments, the log2 fold change (log2fc) of sgRNA counts between before and several days after treatment with the CRISPR-Cas9 system was calculated and normalized for each sgRNA. The normalized log2fc was used for on-target efficiency prediction. 3) The CRISPR-Cas9 dataset used in deepCrispr [32]. It is CRISPR-Cas9 experiment data in four different cell lines (HCT116, HL60, HeLa, and HEK293T). However, these data were collected from both negative selection experiment and positive selection experiment. Around 15,000 sgRNAs were studied and integrated into this dataset. For comparison purpose, the normalized on-target efficiency scores calculated in deepCrispr were also used in our study.

### Feature extraction and preprocessing

#### Sequence feature extraction of off-target dataset

We extracted all base-pairs from each position of aligned sgRNA-DNA sequences. The indices of base-pairs start from 0. The orientation is from 5’ end to 3’ end of a sgRNA. 16 different types of base pairs can form. Specifically, the input sequence length in CRISPR-Cas9 dataset is 20 bases, so 20 base pairs are extracted from a sgRNA-DNA aligned pair. The input sequence length in CRISPR-Cas12a system is 27 bases, so 27 base pairs are extracted from a sgRNA-DNA aligned pair.

#### Sequence feature extraction of on-target dataset

A 2-bases length sliding windows are employed to extract dimers from a sgRNA sequence. The indices of dimers start from 0. The orientation is from 5’ end to 3’ end of sgRNA. 16 different types of dimers can form. For example, AU, UG, GC, and CU are extracted from an AUGCU sequence. To be specific, the input sequence length in CRISPR-Cas9 dataset is 20 bases. 19 dimers are extracted from a sequence. In CRISPR-Cas12a dataset, The input sequence length in CRISPR-Cas12a is 34 bases. 33 dimers are extracted from a sequence in this case.

#### NetExpress score

Cell-specific network-based gene property NetExpress score integrated both gene-gene interaction network information from STRING [69] and gene expression data from Broad Institute Cancer Cell Line Encyclopedia (CCLE) [70] or The Encyclopedia of DNA Element (ENCODE) [30]. NetExpress score was calculated with NEST software. In this method, each gene’s NetExpress score was calculated following these steps: 1) List genes which are connected with the studied gene in the interaction network 2) Calculate the product of the connected gene expression value and gene-gene connection confidence score. Gene-gene connection confidence score is the weight of gene-gene interaction in network 3) Sum all the products to get the NetExpress score.

### Evaluation of off-target specificity prediction model AttnToMismatch_CNN

The implementation details of AttnToMismatch_CNN can be found in S5 Table. The code is available at https://github.com/qiaoliuhub/AttnToCrispr.

#### AttnToMismatch_CNN on CRISPR-Cas12a data

AttnToMismatch_CNN takes a 27-bases long sequence as input and outputs the probability of the sgRNA belonging to either a high activity class or a low activity class. The last part of AttnToMismatch_CNN is a log-softmax function. The loss function for this classification problem is negative log likelihood loss. 5-fold cross-validation was used for model evaluation. We randomly split the dataset into 5 folds. 4 folds were used for the training process, and the remaining data were kept unseen in order to test model performance. This procedure was repeated five times by leaving each fold of data out. The final performance is the average of these five repeats. Evaluation metrics are AUC-ROC and PR-AUC scores. AUC-ROC score is calculated as the area under ROC curve, while PR-AUC score is the area under precision-recall score. The ROC curve is plotted as the true positive rate (TP/(TP+FN)) against the false positive rate (FP/(FP+TN)) under a series of thresholds. The precision-recall curve is plotted as precision (TP/(TP+FP)) versus recall (TP/(TP+FN)) under a series of thresholds.

#### AttnToMismatch_CNN on CRISPR-Cas9 data

AttnToMismatch_CNN takes a 20-bases long sequence as input and outputs the probability of the sgRNA belonging to either a positive sample or a negative sample in this dataset. The loss function for this classification problem is negative log likelihood loss. Both 5-fold cross-validation and leave-3-sgRNAs-out methods are used to evaluate models. i) The details of 5-fold cross-validation were mentioned before. ii) In the leave-3-sgRNAs-out scenario, 3 sgRNAs are kept untouched during the training process. The remaining are used as the training dataset. We have repeated this procedure 10 times. Each time had a different set of sgRNAs as left-out sgRNAs. The metrics used for this classification problem are also AUC-ROC and PR-AUC.

### Evaluation of on-target efficiency prediction models

#### Models on CRISPR-Cas12a data

Models take a 34-bases long sequence as input and output on-target efficiency score, indel frequency in this scenario. The training dataset and test dataset were split as deepCpf1 studies [27]. In that study, the training dataset is labeled as HT 1–1 and has 15,000 sgRNAs. The test dataset is labeled as HT 1–2 and has 1,292 sgRNAs. We used mean squared error as the loss function. The metrics for this regression problem are Pearson correlation and Spearman correlation.

#### Models on CRISPR-Cas9 data

Models take a 20-bases long sequence as input and output on-target efficiency score. Mean squared error is the loss function. Pearson correlation and Spearman correlation are the *evaluation metrics*. i) For dataset in K562, A549, and NB4 with and without NetExpress, 5-fold cross-validation methods were used to evaluate the models. In the study without NetExpress score, this feature was excluded from input features. ii) For dataset in deepCrispr study, data obtained in HCT116, HL60, and HeLa cell lines were from high-throughput negative selection experiment. Data obtained in HEK293T was from positive selection experiment. 1) The test was performed by leaving one cell line’s data out. In this test, three cell lines data were used in the training process, and the rest data were used to test model performance. Each cell line’s data was left out at one time, and four tests were performed. 2) The test was performed with data in HCT116, HL60, and HeLa cell lines and then data in HEK293T cell line only. 5-fold cross-validation method was used.

### Input perturbation based feature importance study

We used input perturbation method to study feature importance in each model [40]. For each feature, we shuffled it across all samples and calculated the eventual losses, mean square error loss for regression problems, and negative log likelihood loss for classification problems. 40 repeated tests were performed for each feature, and average losses were calculated. This average loss score was considered as the raw feature importance of a feature. We then normalized these feature importance using the sum of all features importance.

## Supporting information

S1 Table Comparison of models performance with Spearman correlation, Pearson’s correlation, and mean squared error (MSE) for on-target efficiency prediction of data in K562, A549, and NB4 cell lines. (DOCX)

S2 Table Performance comparison of AttnToCrispr_CNN with deepCRISPR. The test was performed following a 5-fold cross-validation procedure.
(DOCX)

S3 Table Model performances of positive experiment data in HEK293T cell line with 5-fold cross-validation. (DOCX)

S4 Table Performance comparison of models with more cell-specific features and without these features for on-target efficiency prediction of data in K562, A549, and NB4 cell lines. These cell-specific features include DNase-seq, Chip-seq for CTCF, and H3K4me3 and RRBS data.
(DOCX)

S5 Table Hyperparameters of each component in the implemented models. (DOCX)

S1 Note Explanation on the performance differences of on-target efficiency predictions of CRISPR-Cpf1 dataset and CRISPR-Cas9 dataset. (DOCX)
