# Supplementary material for: Prediction of off-target specificity and cell-specific fitness of CRISPR-Cas System using attention boosted deep learning and network-based gene feature
Source: PLoS Comput Biol. 2019 Oct 28;15(10):e1007480. doi: 10.1371/journal.pcbi.1007480 (PMC6837542; doi:10.1371/journal.pcbi.1007480)
Supplement: S1 Table — (DOCX) [file pcbi.1007480.s001.docx]

**S1 Table.** Comparison of models performance with Spearman correlation, Pearson’s correlation, and mean squared error (MSE) for on-target efficiency prediction of data in K562, A549, and NB4 cell lines.

| **Dataset** | **Model** | **Spearman** | **Pearson** | **MSE** |
| --- | --- | --- | --- | --- |
| **K562** | **Random Forest** | 0.395 ± 0.000 | 0.386 ± 0.001 | 0.405 ± 0.001 |
|  | **Gradient Boosted Trees** | 0.388 ± 0.000 | 0.348 ± 0.000 | 0.419 ± 0.002 |
|  | **seqCrispr** | 0.414 ± 0.003 | 0.383 ± 0.001 | 0.401 ± 0.003 |
|  | **attnToCrispr_CNN** | **0.437 ± 0.000** | **0.395 ± 0.000** | **0.397 ± 0.002** |
| **K562_NetExp** | **Random Forest** | 0.412± 0.001 | 0.429 ± 0.000 | 0.386 ± 0.002 |
|  | **Gradient Boosted Trees** | 0.406 ± 0.000 | 0.419 ± 0.000 | 0.394 ± 0.001 |
|  | **seqCrispr** | 0.462 ± 0.003 | 0.456 ± 0.002 | 0.369 ± 0.003 |
|  | **attnToCrispr_CNN** | **0.468 ± 0.000** | **0.460 ± 0.000** | **0.374 ± 0.002** |
| **A549** | **Random Forest** | 0.382 ± 0.000 | 0.395 ± 0.000 | 0.341 ± 0.001 |
|  | **Gradient Boosted Trees** | 0.355 ± 0.000 | 0.357 ± 0.000 | 0.357 ± 0.002 |
|  | **seqCrispr** | 0.386 ± 0.002 | 0.391 ± 0.001 | 0.343 ± 0.002 |
|  | **attnToCrispr_CNN** | **0.406 ± 0.000** | **0.422 ± 0.000** | **0.328 ± 0.001** |
| **A549_NetExp** | **Random Forest** | 0.451 ± 0.000 | 0.453 ± 0.000 | 0.319 ± 0.002 |
|  | **Gradient Boosted Trees** | 0.461 ± 0.000 | 0.501 ± 0.000 | 0.300 ± 0.002 |
|  | **seqCrispr** | 0.463 ± 0.002 | 0.483 ± 0.002 | 0.302 ± 0.002 |
|  | **attnToCrispr_CNN** | **0.476 ± 0.000** | **0.513 ± 0.000** | **0.295 ± 0.001** |
| **NB4** | **Random Forest** | 0.373 ± 0.000 | 0.382 ± 0.000 | 0.163 ± 0.001 |
|  | **Gradient Boosted Trees** | 0.323 ± 0.000 | 0.332 ± 0.000 | 0.168 ± 0.001 |
|  | **seqCrispr** | 0.395 ± 0.003 | 0.399 ± 0.002 | 0.161 ± 0.001 |
|  | **attnToCrispr_CNN** | **0.392 ± 0.000** | **0.395 ± 0.000** | **0.160 ± 0.001** |
| **NB4_NetExp** | **Random Forest** | 0.399 ± 0.000 | 0.479 ± 0.000 | 0.149 ± 0.001 |
|  | **Gradient Boosted Trees** | 0.364 ± 0.000 | 0.394 ± 0.000 | 0.164 ± 0.001 |
|  | **seqCrispr** | 0.431 ± 0.003 | 0.445 ± 0.001 | 0.153 ± 0.002 |
|  | **attnToCrispr_CNN** | **0.439 ± 0.000** | **0.463 ± 0.000** | **0.148 ± 0.001** |
