# Supplementary material for: Prediction of off-target specificity and cell-specific fitness of CRISPR-Cas System using attention boosted deep learning and network-based gene feature
Source: PLoS Comput Biol. 2019 Oct 28;15(10):e1007480. doi: 10.1371/journal.pcbi.1007480 (PMC6837542; doi:10.1371/journal.pcbi.1007480)
Supplement: S2 Table — The test was performed following a 5-fold cross-validation procedure. (DOCX) [file pcbi.1007480.s002.docx]

**S2 Table.** Performance comparison of AttnToCrispr_CNN with deepCRISPR. The test was performed following a 5-fold cross-validation procedure.

|  | Cell line | model | Spearman | Pearson | MSE |
| --- | --- | --- | --- | --- | --- |
| deepCrispr  (5 fold cv)  <https://github.com/bm2-lab/DeepCRISPR/blob/master/paper_data-regression.tar.gz> * | HL60 | **deepCrispr** | 0.262 |  |  |
|  |  | **attnToCrispr_CNN** | **0.274** | 0.311 | 0.013 |
|  | HCT116 | **deepCrispr** | 0.654 |  |  |
|  |  | **attnToCrispr_CNN** | **0.752** | 0.756 | 0.013 |
|  | HeLa | **deepCrispr** | 0.501 |  |  |
|  |  | **attnToCrispr_CNN** | **0.573** | 0.572 | 0.022 |
|  | HEK293T | **deepCrispr** | **0.871** |  |  |
|  |  | **attnToCrispr_CNN** | 0.824 | 0.846 | 0.003 |
|  | All | **deepCrispr** | 0.601 | - | - |
|  |  | **attnToCrispr_CNN** | **0.650** | 0.651 | 0.015 |
| deepCrispr  (5 fold cv)  Supplementary data * | HL60 | **deepCrispr** | 0.262 |  |  |
|  |  | **attnToCrispr_CNN** | **0.275** | 0.323 | 0.016 |
|  | HCT116 | **deepCrispr** | 0.654 |  |  |
|  |  | **attnToCrispr_CNN** | **0.707** | 0.728 | 0.014 |
|  | HeLa | **deepCrispr** | 0.501 |  |  |
|  |  | **attnToCrispr_CNN** | **0.561** | 0.568 | 0.023 |
|  | HEK293T | **deepCrispr** | **0.871** |  |  |
|  |  | **attnToCrispr_CNN** | 0.081 | 0.086 | 0.020 |
|  | All | **deepCrispr** | 0.601 | - | - |
|  |  | **attnToCrispr_CNN** | **0.560** | 0.651 | 0.015 |

***** We found different versions of datasets used in deepCRISPR from two resources, one from <https://github.com/bm2-lab/DeepCRISPR/blob/master/paper_data-regression.tar.gz> and the other from paper’s supplementary section. Performance comparison was conducted with both versions of data.
