# Supplementary material for: Prediction of off-target specificity and cell-specific fitness of CRISPR-Cas System using attention boosted deep learning and network-based gene feature
Source: PLoS Comput Biol. 2019 Oct 28;15(10):e1007480. doi: 10.1371/journal.pcbi.1007480 (PMC6837542; doi:10.1371/journal.pcbi.1007480)
Supplement: S3 Table — (DOCX) [file pcbi.1007480.s003.docx]

**S3 Table:** Model performances of positive experiment data in HEK293T cell line with 5-fold cross-validation.

|  | Cell line | model | Spearman | Pearson | MSE |
| --- | --- | --- | --- | --- | --- |
| HEK293T  (5 fold cv) | HEK293T | **deepCrispr** | **0.871** |  |  |
|  |  | **attnToCrispr_CNN** | 0.234 | 0.262 | 0.0135 |
