# Supplementary material for: Prediction of off-target specificity and cell-specific fitness of CRISPR-Cas System using attention boosted deep learning and network-based gene feature
Source: PLoS Comput Biol. 2019 Oct 28;15(10):e1007480. doi: 10.1371/journal.pcbi.1007480 (PMC6837542; doi:10.1371/journal.pcbi.1007480)
Supplement: S4 Table — These cell-specific features include DNase-seq, Chip-seq for CTCF, and H3K4me3 and RRBS data. (DOCX) [file pcbi.1007480.s004.docx]

**S4 Table.** Performance comparison of models with more cell-specific local genetic features and without these features for on-target efficiency prediction of data in K562, A549, and NB4 cell lines. These cell-specific local genetic features include DNase-seq, Chip-seq for CTCF, and H3K4me3 and RRBS data. The performance metric is spearman correlation.

| **Cell line** | **with extra features** | **without extra features** |
| --- | --- | --- |
| K562 | 0.463 ± 0.000 | 0.468 ± 0.000 |
| A549 | 0.476 ± 0.000 | 0.476 ± 0.000 |
| NB4 | 0.435 ± 0.000 | 0.439 ± 0.000 |
