# Supplementary material for: Prediction of off-target specificity and cell-specific fitness of CRISPR-Cas System using attention boosted deep learning and network-based gene feature
Source: PLoS Comput Biol. 2019 Oct 28;15(10):e1007480. doi: 10.1371/journal.pcbi.1007480 (PMC6837542; doi:10.1371/journal.pcbi.1007480)
Supplement: S5 Table — (DOCX) [file pcbi.1007480.s005.docx]

**S5 Table**. Hyperparameters of each component in the implemented models

|  |  | AttnTtoMismatch_CNN | | AttnToCrispr_CNN | |
| --- | --- | --- | --- | --- | --- |
|  |  | CRISPR-Cas12a | CRSPR-Cas9 | CRISPR-Cas12a | CRSPR-Cas9 |
| Embedding Layer | Embedding dimension | 8 | 8 | 64 | 64 |
|  | Embedding output dimension | 27 x 8 | 23 x 8 | 34 x 64 | 23 x 64 |
| Transformer | Embedding dimension | 8 | 8 | 64 | 64 |
|  | Feed-forward hidden unit | 32 | 32 | 256 | 256 |
|  | Number of heads | 2 | 2 | 16 | 16 |
|  | Output dimension | 27 x 8 | 23 x 8 | 34 x 64 | 23 x 64 |
| CNN | Conv2d kernel size | [(3,1), (3,1)] | [(3,1), (3,1)] | [(3,1), (3,1)] | [(3,1), (3,1)] |
|  | Max pooling kernel size | [(2,1),(2,1)] | [(2,1),(2,1)] | [(2,1),(2,1)] | [(2,1),(2,1)] |
|  | Number of layers | 2 | 2 | 2 | 2 |
| Fully connected | Number of hidden unit | 100 | 100 | [2000, 40] | [2000, 40] |
| Dropout | | 0.5 | 0.5 | 0.3 | 0.2 |
| Learning rate | | 0.0003 | 0.0003 | 0.0003 | 0.0003 |
