# Supplementary material for: Prediction of off-target specificity and cell-specific fitness of CRISPR-Cas System using attention boosted deep learning and network-based gene feature
Source: PLoS Comput Biol. 2019 Oct 28;15(10):e1007480. doi: 10.1371/journal.pcbi.1007480 (PMC6837542; doi:10.1371/journal.pcbi.1007480)
Supplement: S1 Note — (DOCX) [file pcbi.1007480.s006.docx]

**S1 Note**

Performances of on-target efficiency predictions of CRISPR-Cpf1 dataset and CRISPR-Cas9 dataset are different. It is hard to draw a conclusion regarding this difference. Kim et al. proposed two reasons [1]. i) deepCpf1 dataset is larger than the dataset they used for CRISPR-Cas9. However, this reason can be ruled out with our data because the CRISPR-Cas9 data size is larger than deepCpf1 dataset. ii) Kim et al. suggested that indel frequency were scarcely affected by chromatin accessibility and less noisy because sgRNAs targeted integrated sites in this dataset, while they targeted target sites in the endogenous genome. However, a more conclusive statement needs more data to support.

1. Kim HK, Min S, Song M, Jung S, Choi JW, Kim Y, et al. Deep learning improves prediction of CRISPR-Cpf1 guide RNA activity. Nat Biotechnol. 2018;36(3):239-41. Epub 2018/02/13. doi: 10.1038/nbt.4061. PubMed PMID: 29431740.
